# Prognostic Value of Coronary Angiography‐Derived Index of Microvascular Resistance in Patients With Hypertrophic Cardiomyopathy

**DOI:** 10.1002/mco2.70289

**Published:** 2025-07-25

**Authors:** Yuxuan Zhang, Rui Ji, Shuxin Lei, Jingnan Pan, Zining Chen, Shitian Guo, Delong Chen, Abuduwufuer Yidilisi, Jiacheng Fang, Yiyue Zheng, Xinyi Zhang, Chi Liu, Jiniu Huang, Yumeng Hu, Jianping Xiang, Xiaojie Xie, Jian'an Wang, Jun Jiang

**Affiliations:** ^1^ Department of Cardiology of The Second Affiliated Hospital School of Medicine, Zhejiang University Hangzhou China; ^2^ State Key Laboratory of Transvascular Implantation Devices Hangzhou China; ^3^ Cardiovascular Key Laboratory Zhejiang Province Hangzhou China; ^4^ Transvascular Implant Instrument Research Institute The Second Affiliated Hospital Zhejiang University School of Medicine, Binjiang District Hang Zhou China; ^5^ Department of Cardiology Ningbo Medical Center Lihuili Hospital, Ningbo University Ningbo Zhejiang China; ^6^ ArteryFlow Technology Co., Ltd. Hangzhou China

**Keywords:** coronary angiography, hypertrophic cardiomyopathy, index of microcirculatory resistance, microcirculation, prognosis

## Abstract

Assessing coronary microcirculation is crucial in the progression of hypertrophic cardiomyopathy (HCM), but it's often inadequate in clinical practice. This study investigates the role of coronary microcirculation, assessed via angiography‐derived index of microvascular resistance (angio‐IMR), in predicting clinical outcomes in HCM patients. We retrospectively measured angio‐IMR in 422 HCM patients across two sites. The primary endpoint was major advance cardiovascular event (MACE), including cardiovascular death, heart failure readmission, life‐threatening ventricular arrhythmias, septal reduction therapy or new‐onset stroke. Over a mean follow‐up of 43 ± 23 months, 63 patients (14.93%) experienced MACE. The mean angio‐IMR value for the left anterior descending artery (LAD) was 22 ± 8. Using maximally selected log‐rank statistic, 123 patients were stratified into the high LAD angio‐IMR group, indicating microvascular dysfunction. Patients with LAD angio‐IMR > 25 exhibited a higher incidence of MACE than those with LAD angio‐IMR ≤ 25 (25.4% vs. 13.3%, *p* = 0.035). After adjusting for risk factors, elevated LAD angio‐IMR remained an independent predictor of MACE (HR 1.779, 95% CI, 1.053–3.007, *p* = 0.031). Subgroup analysis showed consistent results. Our findings underscore that elevated LAD angio‐IMR is a robust, independent indicator of adverse prognosis in HCM patients, highlighting the importance of evaluating LAD angio‐IMR during coronary angiography for these patients.

## Graphical abstract

With an angio‐IMR threshold of over 25, nearly one‐third of HCM patients demonstrated CMD in the LAD. Patients with LAD angio‐IMR > 25 exhibited a higher incidence of MACE compared to those with LAD angio‐IMR ≤ 25 (cumulative incidence, 25.4% vs. 13.3%, *p* = 0.035). In HCM patients who underwent coronary angiography without stenting, angio‐IMR could be used as an additional risk stratification and precise assessment tool.

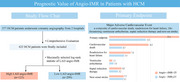

## 1 Introduction

Hypertrophic cardiomyopathy (HCM) is a prevalent genetic cardiovascular disease worldwide, with a reported prevalence of 1/500–1/200, which may be underestimated [1, 2]. Due to the development and progress in clinical management, the overall mortality rate of HCM patients has been greatly improved compared to the past [3]. However, 10% of patients will still experience two or more complications in their lifetime, imposing a significant burden on both the patients and society [4, 5].

Coronary microvascular dysfunction (CMD), a key pathophysiological feature in HCM, occurs not only in hypertrophied but also in nonhypertrophied left ventricular (LV) segments, even without significant epicardial coronary artery stenosis (CAD) [1, 6, 7]. The pathogenesis of CMD appears to be multifactorial, encompassing factors such as decreased capillary density, vascular remodeling characterized by arteriolar medial hypertrophy and intimal hyperplasia, fibrosis, myocyte disarray, extravascular compression resulting from ventricular hypertrophy, diastolic dysfunction, and LV outflow tract (LVOT) obstruction [8].

Moreover, the presence of CMD, whether identified through histology examination or assessed with imaging techniques, has been shown to be a key factor leading to the progression of the syndrome and adverse outcomes, including increased risks of ventricular hypertrophy, arrhythmias, and heart failure [8, 9, 10, 11, 12]. Therefore, for the management of patients with HCM, assessing and treating CMD is an important strategy to improve prognosis.

Research has shown that commonly used assessment methods for CMD, including noninvasive techniques such as positron emission tomography (PET) and cardiovascular magnetic resonance (CMR), and invasive approaches like coronary flow reserve (CFR) and the index of microvascular resistance (IMR), are effective in evaluating CMD in HCM patients [9, 10, 11, 13, 14]. In these technologies, researches are primarily focused on noninvasive assessment methods such as PET and CMR. Large‐scale clinical studies have confirmed that the CMD assessed by these methods is an independent risk factor for poor prognosis in HCM patients [13, 15, 16]. These researches have provided new insights into the treatment and management of HCM and has also encouraged some clinicians to use these techniques to evaluate HCM patients. However, these methods are still not routinely included in the clinical assessment of HCM patients due to the complexity of the procedures, the need for vasodilator drugs, and the cost implications. Especially CFR and IMR, which have been proven to be effective in assessing coronary microcirculation in patients with CAD, are rarely used to evaluate CMD in HCM patients due to their invasiveness and reliance on special guidewires [17].

In recent years, the innovation of angiography‐derived IMR (angio‐IMR) offers a new approach to swiftly calculate invasive IMR based on angiographic images without wires, which facilitates the assessment of patients’ coronary microcirculation. The angio‐IMR has demonstrated strong concordance with invasive IMR and has been proven to have clinical prognostic value in patients with chronic coronary syndrome, acute ST‐segment elevation myocardial infarction, acute non‐ST‐segment elevation myocardial infarction and heart failure with preserved ejection fraction [18, 19, 20, 21, 22]. Currently, there is a lack of clinical data on the use of angio‐IMR to assess the microcirculation status and long‐term prognostic value in HCM patients. This study sought to characterize coronary microvascular function in HCM patients through angio‐IMR measurement and to explore how CMD influences long‐term clinical outcomes.

## 2 Results

### 2.1 Baseline Traits and Cohort Assignments

**FIGURE 1:**
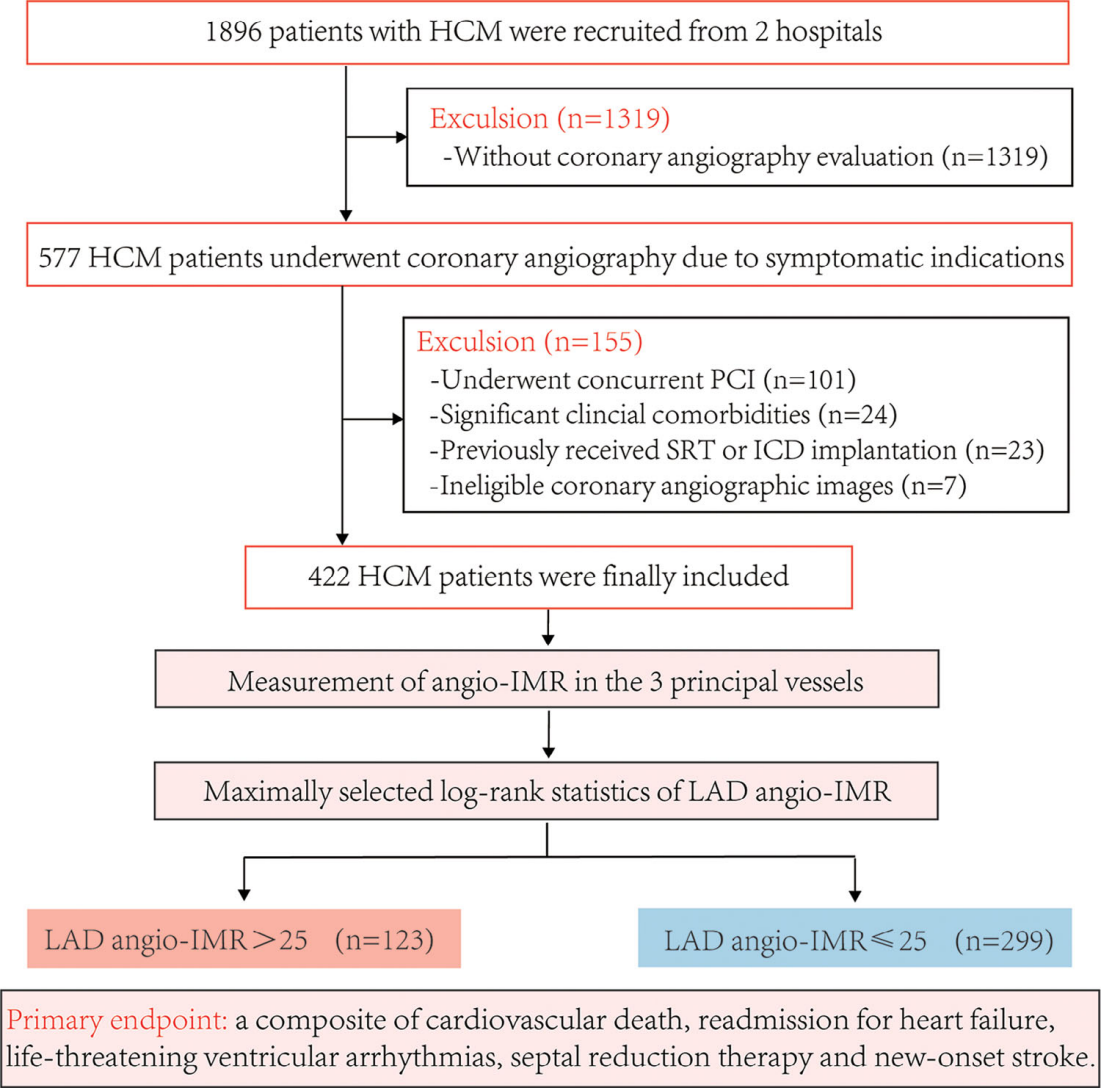
Study flow. Angio‐IMR, angiography‐derived index of microvascular resistance; HCM, hypertrophic cardiomyopathy; ICD, implantable cardioverter defibrillator; LAD, left anterior descending artery; PCI, percutaneous coronary intervention; SRT, septal reduction therapy.

Initially, 1896 consecutive HCM patients from two hospitals were screened, with 557 proceeding to symptom‐driven coronary angiography, and 422 were retained for study participation following application of exclusion criteria (Figure 1). The excluded patients consisted of 101 individuals who had concurrent percutaneous coronary intervention (PCI), 24 with significant clinical comorbidities, 23 who had undergone septal reduction therapy (SRT) or received an implantable cardioverter‐defibrillator (ICD) implantation, and seven with ineligible coronary angiographic images (Figure 1). Table 1 details the baseline clinical parameters of this study cohort, indicating a mean age of 60 ± 11 years, a BMI of 25.3 ± 3.3 kg/m^2^. Smoking was reported in 139 individuals (32.9%), while alcohol consumption was noted in 97 (23.0%). The majority of the patients (76.6%) had New York Heart Association (NYHA) functional class I–II, with only a subset (29.1%) presenting with LVOT obstruction, collectively suggesting a relatively low disease burden among the enrolled patients. In terms of cardiovascular risk factors, over half of the patients have comorbid hypertension (53.6%), nearly one‐third have dyslipidemia (37.4%), and a small proportion have diabetes mellitus (15.6%) and CAD (23.7%). After completing the angio‐IMR measurements of the 3 principal vessels, it was found that the angio‐IMR was 22 ± 8 in the left anterior descending artery (LAD), 20 ± 6 in the left circumflex artery (LCX), and 26 ± 9 in the right coronary artery (RCA).

**TABLE 1:** Baseline clinical characteristics of included patients.

	Total (*n* = 422)	High angio‐IMR (*n* = 123)	Low angio‐IMR (*n* = 299)	*p* value
Demographics				
Age, year	60 ± 11	58 ± 12	61 ± 11	0.059
Male sex, *n* (%)	250 (59.2)	89 (72.4)	161 (53.8)	< 0.001
BMI, kg/m^2^	25.3 ± 3.3	25.6 ± 3.5	25.2 ± 3.2	0.207
Smoking, *n* (%)	139 (32.9)	45 (36.6)	94 (31.4)	0.307
Alcohol intake, *n* (%)	97 (23.0)	41 (33.3)	56 (18.7)	0.001
NYHA functional class, no. (%)				0.638
I	115 (27.3)	37 (30.1)	78 (26.1)	
II	208 (49.3)	55 (44.7)	153 (51.2)	
III	77 (18.2)	25 (20.3)	52 (17.4)	
IV	22 (5.2)	6 (4.9)	16 (5.4)	
Cardiovascular risk factors, *n* (%)				
Diabetes mellitus	66 (15.6)	21 (17.1)	45 (15.1)	0.603
Hypertension	226 (53.6)	64 (52.0)	162 (54.2)	0.688
Dyslipidemia	158 (37.4)	50 (40.7)	108 (36.1)	0.382
CAD	100 (23.7)	30 (24.4)	70 (23.4)	0.830
Family history, *n* (%)				
Hypertrophic cardiomyopathy	13 (3.1)	5 (4.1)	8 (2.7)	0.659
Sudden death from cardiac causes	9 (2.1)	2 (1.6)	7 (2.3)	0.927
Echocardiographic indices				
LVOT obstruction (≥ 30 mm Hg), *n* (%)	123 (29.1)	28 (22.8)	95 (31.8)	0.064
AO‐STJ	2.91 ± 0.36	2.92 ± 0.31	2.90 ± 0.38	0.529
EF	67 ± 8	65 ± 9	67 ± 8	0.015
IVSd	1.55 ± 0.46	1.54 ± 0.47	1.55 ± 0.46	0.797
IVSs	1.86 ± 0.42	1.90 ± 0.44	1.84 ± 0.41	0.184
LVIDd	4.46 ± 0.57	4.58 ± 0.61	4.42 ± 0.55	0.010
LVIDs	2.81 ± 0.48	2.93 ± 0.55	2.76 ± 0.44	0.001
LVM	233.56 ± 83.69	239.96 ± 83.29	230.92 ± 83.85	0.314
Angiography‐derived indices				
LAD				
Angio‐FFR	0.91 ± 0.06	0.95 ± 0.03	0.89 ± 0.06	< 0.001
Angio‐IMR	22 ± 8	31 ± 5	18 ± 4	< 0.001
MB, *n* (%)	97 (23.0)	26 (21.1)	71 (23.7)	0.563
LCX				
Angio‐FFR	0.94 ± 0.03	0.96 ± 0.02	0.94 ± 0.04	< 0.001
Angio‐IMR	20 ± 6	24 ± 18	18 ± 5	< 0.001
RCA				
Angio‐FFR	0.94 ± 0.03	0.96 ± 0.04	0.94 ± 0.03	< 0.001
Angio‐IMR	26 ± 9	31 ± 10	23 ± 7	< 0.001
All				
Angio‐IMR	67 ± 18	86 ± 15	60 ± 13	< 0.001
Laboratory data				
Glucose	5.36 ± 1.39	5.43 ± 1.32	5.33 ± 1.42	0.510
Hemoglobin A1c	6.15 ± 0.98	6.14 ± 1.00	6.18 ± 0.95	0.707
Cholesterol	4.35 ± 1.04	4.37 ± 0.97	4.35 ± 1.07	0.876
High‐density lipoprotein	1.16 ± 0.28	1.12 ± 0.24	1.18 ± 0.30	0.068
Low‐density lipoprotein	2.38 ± 0.77	2.39 ± 0.73	2.48 ± 0.78	0.925
Creatinine	71.37 ± 17.51	74.09 ± 18.69	70.25 ± 16.90	0.041
Troponin T	0.02 (0.01–0.04)	0.02 (0.01–0.04)	0.02 (0.01–0.04)	0.456
B‐type natriuretic peptide	144.35 (62.88–355.53)	133.70 (42.20–352.80)	152.00 (67.60–367.60)	0.302
N‐terminal pro‐BNP	582.00 (170.00–1572.00)	440.00 (109.25–1476.50)	641.00 (236.00–1658.75)	0.311
Medical treatment				
βedical t	343 (81.3)	235 (78.6)	108 (87.8)	0.028
CCB	147 (34.8)	36 (29.3)	111 (37.1)	0.124
ACEI/ARB	183 (43.4)	57 (46.3)	126 (42.1)	0.429
Amiodarone	19 (4.5)	6 (4.9)	13 (4.3)	0.811
Antiplatelet	261 (61.8)	76 (61.8)	185 (61.9)	0.987
Anticoagulant	48 (11.4)	19 (15.4)	29 (9.7)	0.091
Statin	325 (77.0)	90 (73.2)	235 (78.6)	0.229

Abbreviations: ACEI, angiotensin‐converting enzyme inhibitor; angio‐FFR, angiography‐derived fractional flow reserve; angio‐IMR, angiography‐derived index of microvascular resistance; AO‐STJ, aorta to sinutubular junction; ARB, angiotensin receptor blocker; BMI, body mass index; CAD, coronary artery disease; CCB, calcium channel blocker; EF, ejection fraction; IVSd, interventricular septum diastolic thickness; IVSs, interventricular septum systolic thickness; LVIDd, left ventricular internal diameter in diastole; LVIDs, left ventricular internal diameter in systole; LVM, left ventricular mass; LVOT, left ventricular outflow tract; MB, myocardial bridge; NYHA, New York Heart Association. Continuous variables were expressed as mean ± standard deviation (SD) or median (interquartile range [IQR]), while categorical variables were summarized as frequencies and percentages.

**FIGURE 2:**
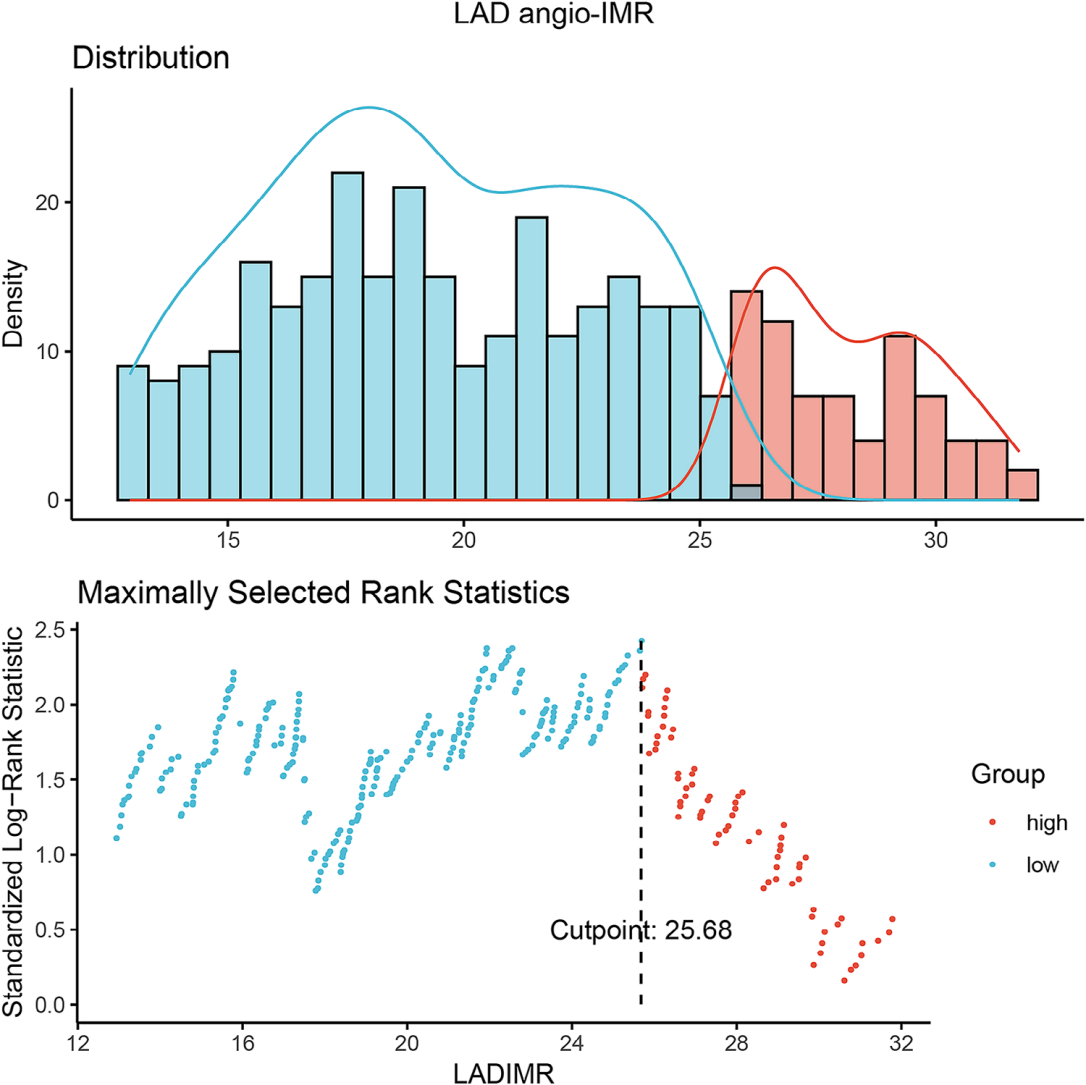
Maximally selected log‐rank statistics of LAD angio‐IMR. Abbreviations as in Figure 1.

Considering the predominant localization of hypertrophy in patients with HCM occurs in the interventricular septum, with a focus on the basal segment, we utilized the LAD angio‐IMR for stratified analysis of the cohort. The cut‐off for the LAD angio‐IMR was established at 25.68 by the application of maximally selected log‐rank statistics (Figure 2). For pragmatic clinical purposes, we adopted an LAD angio‐IMR threshold of 25, categorizing 123 patients with LAD angio‐IMR > 25 and 299 with LAD angio‐IMR ≤ 25. Subtle variations were identified in baseline parameters between patients with elevated LAD angio‐IMR and those with reduced values, particularly regarding gender, alcohol consumption history, echocardiographic findings, such as ejection fraction, LV internal diameter in diastole and LV internal diameter in systole, and angiography‐derived indices (Table 1).

### 2.2 Angio‐IMR's Prognostic Role in HCM Prognosis

**FIGURE 3:**
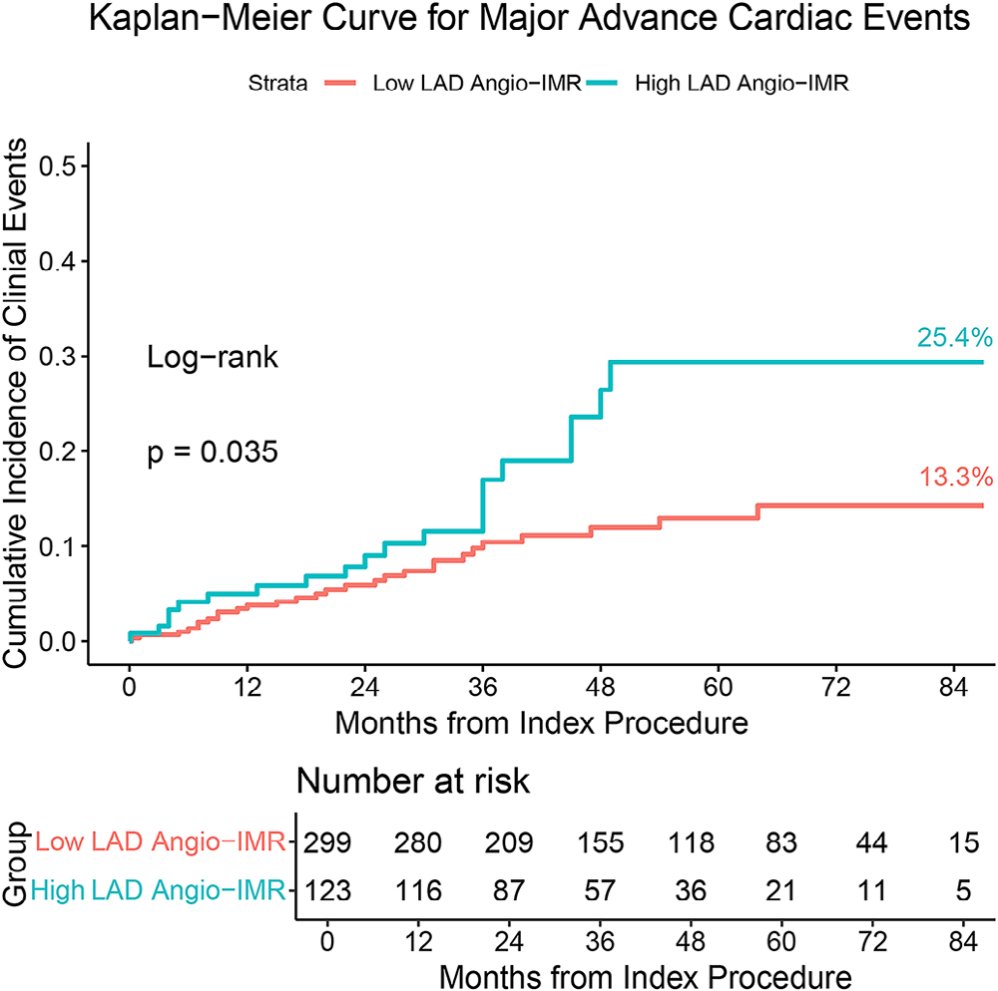
Primary clinical outcomes in HCM patients according to LAD angio‐IMR. CI, confidence interval; HR, hazard ratio; other abbreviations as in Figure 1.

Major adverse cardiovascular events (MACE) were observed in 63 patients (14.93%) over a mean follow‐up period of 43 + 23 months. Throughout the follow‐up period, patients with elevated LAD angio‐IMR exhibited a significantly higher incidence of MACE compared to those with lower values (cumulative incidence at 84 months, 25.4% vs. 13.3%, *p* = 0.035, Table 2 and Figure 3). Upon dissecting the individual components of MACE, it became evident that the observed disparity in MACE rates between groups was predominantly driven by disparities in mortality, particularly cardiac death (cumulative incidence at 84 months, 14.3% vs. 4.3%, *p* = 0.005, Table 2). During the follow‐up period, there were no significant differences between the two groups in the incidence of heart failure readmission, life‐threatening ventricular arrhythmias requiring ICD implantation, SRT, or new‐onset stroke (Table 2).

**TABLE 2:** Clinical outcomes in HCM patients stratified by LAD angio‐IMR.

	High angio‐IMR, *n* = 123 (cumulative incidence)	Low angio‐IMR, *n* = 299 (cumulative incidence)	*p* value
MACE, *n* (%)	25 (25.4)	38 (13.3)	0.035
Death from cardiovascular causes, *n* (%)	11 (14.3)	8 (4.7)	0.010
Readmission for heart failure, *n* (%)	8 (7.8)	13 (5.4)	0.359
Life‐threatening ventricular arrhythmias requires an ICD implantation, *n* (%)	1 (1.1)	3 (1.6)	0.858
Septal myocardial ablation or septal myectomy, *n* (%)	4 (4.5)	8 (3.2)	0.564
New‐onset stroke, *n* (%)	6 (5.3)	11 (5.1)	0.843
Cardiac death, *n* (%)	11 (14.3)	7 (4.3)	0.005

Abbreviations: ICD, implantable cardioverter defibrillator; MACE, major adverse cardiovascular event; other abbreviations as in Table 1.

In the univariate regression analysis, LAD angio‐IMR > 25 was identified as a risk factor for MACE in HCM patients (HR: 1.704, 95% CI, 1.028–2.825, *p* = 0.039, Tables 3 and S1). After adjusting for covariables, multivariate regression analysis corroborated that high LAD angio‐IMR persisted as an independent predictor of MACE in patients with HCM (HR: 1.779, 95% CI, 1.053–3.007, *p* = 0.031, Table 3). Other independent risk factors include age and high LV mass (Table 3).

**TABLE 3:** Cox proportional survival analysis for MACE in HCM patients.

Covariates	Univariable analysis		Multivariable analysis	
	HR (95% CI)	*p* value	HR (95% CI)	*p* value
LAD angio‐IMR > 25	1.704 (1.028–2.825)	0.039	1.779 (1.053–3.007)	0.031
Age, per 10 years	1.394 (1.109–1.751)	0.004	1.455 (1.135–1.866)	0.003
NYHA functional class III or IV	1.697 (1.014–2.840)	0.044		
EF < 50	4.169 (1.510–11.510)	0.006		
LV mass > 262	1.654 (1.000–2.735)	0.0499	1.786 (1.069–2.984)	0.027
β‐Blocker	0.865 (0.470–1.593)	0.642		
Antiplatelet	0.654 (0.397–1.075)	0.094		
Anticoagulant	4.196 (2.416–7.289)	0.008		

Abbreviations: CI, confidence interval; HR, hazard ratio; other abbreviations as in Table 1.

### 2.3 Subgroup Analysis and PSM Analysis

**FIGURE 4:**
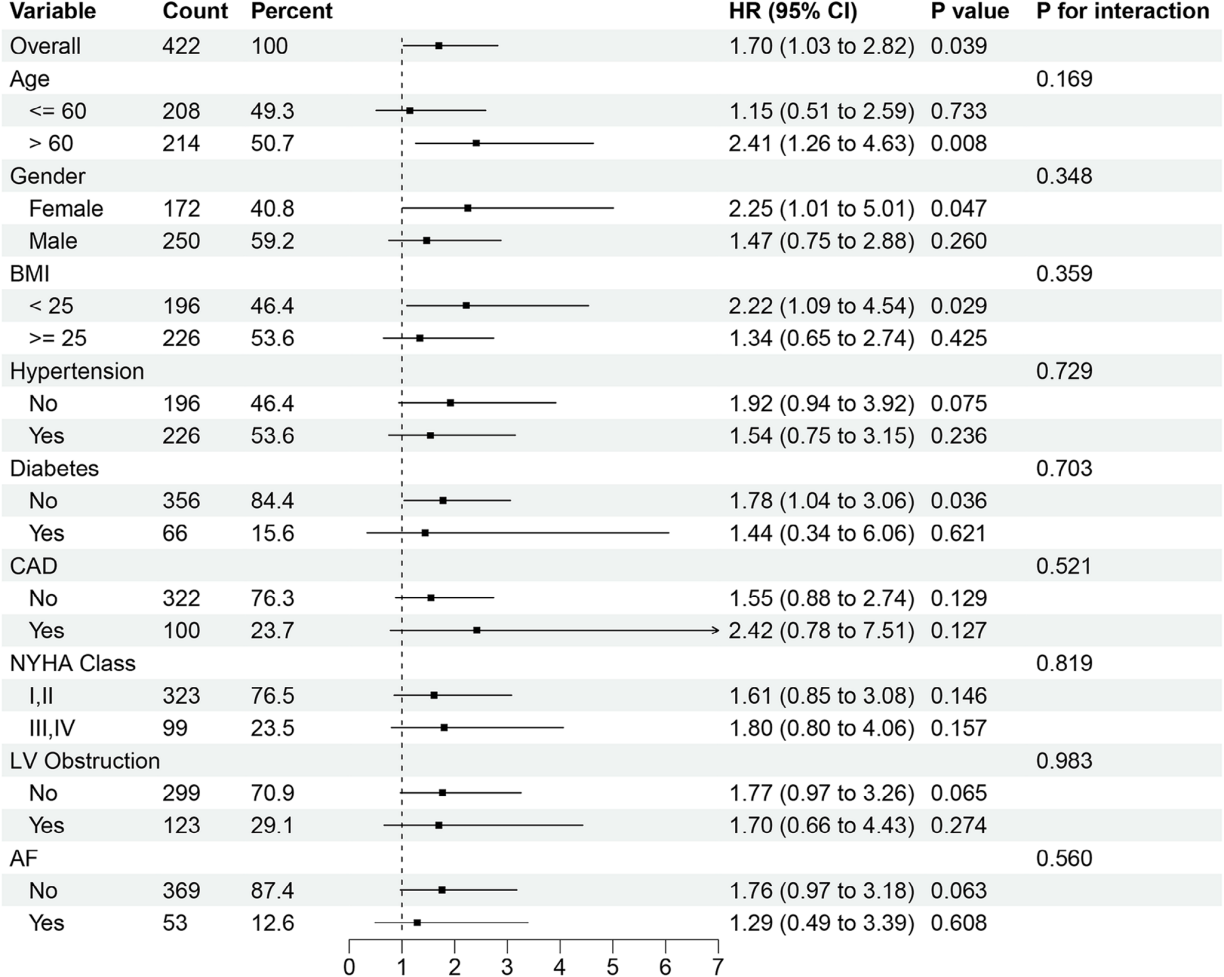
Subgroup analysis of the impact of LAD angio‐IMR on MACE across various subgroups. AF, atrial fibrillation; BMI, body mass index; CAD, coronary artery disease; LV, left ventricular; NYHA, New York Heart Association; other abbreviations as in Figure 3.

Subgroup analysis revealed consistent prognostic effects of LAD angio‐IMR across different patient categories: patients ≦ 60 years of age and those > 60 years, female and male, those BMI < 25 and those ≧25, those baseline with cardiovascular risk factors at baseline and those without, those with NYHA functional class I–II and those with III–IV and those with LVOT obstruction and those without (Figure 4).

Considering the baseline disparities in HCM patients with and without CMD in LAD territory, PSM was performed using a 1:1 nearest neighbor algorithm with a caliper width of 0.2. The matching process incorporated covariates including age, male sex, alcohol intake, NYHA functional class, echocardiographic indices, angio‐FFR, and discharge medications. Following PSM, baseline characteristics achieved balance between groups (Table S2). The results of the Kaplan–Meier survival curve analysis were consistent with the aforementioned findings, showing that HCM patients with high LAD angio‐IMR associated with elevated MACE incidence during follow‐up (Figure S1).

### 2.4 Sensitivity Analysis

We conducted a sensitivity analysis to assess the impact of the total angio‐IMR (the sum of three vessels’ angio‐IMR), the LCX angio‐IMR, and the RCA angio‐IMR on patient prognosis. In line with clinical practice, we adopted a threshold of 25 for both LCX angio‐IMR and RCA angio‐IMR, and thereby a threshold of 75 for total angio‐IMR. When stratified by total angio‐IMR, it was observed that patients with elevated total angio‐IMR exhibited a higher incidence of MACE compared to those with lower values, although the disparity was not statistically significant (cumulative incidence at 84 months, 20.4% vs. 15.1%, *p* = 0.480, Figure S2). Nonetheless, stratification based on either LCX angio‐IMR or RCA angio‐IMR failed to reveal any substantial differences in MACE incidence between the groups (Figure S2).

## 3 Discussion

This is a large‐scale cohort study that pioneers the use of angio‐IMR in evaluating coronary microcirculation among HCM patients, and for the first time, establishes a link between CMD in the LAD zone and long‐term prognosis. The central discoveries of this study as follow: (1) With an angio‐IMR threshold of over 25, nearly one‐third of HCM patients demonstrated CMD in the LAD; (2) In HCM patients, CMD localized to LAD zone is associated with a significantly increased MACE incidence, with LAD angio‐IMR > 25 emerged as an independent risk factor, highlighting the prognostic impact of CMD in the LAD zone is particularly pronounced compared to other vessels. These findings underscore the significance of calculating the LAD angio‐IMR during coronary angiography for HCM patients.

In HCM, CMD emerged from the intricate interplay between ultrastructural abnormalities and hemodynamic factors that compromise the microvascular system [15, 23]. The ultrastructural abnormalities encompass disorganized cardiomyocyte architecture, diminished capillary density, arterial remodeling, and vascular fibrosis [23, 24, 25]. These phenomena may be explained by the capillary: myocyte coupling hypothesis, which posits that the microvasculature and matrix meshwork are abnormal even during organogenesis [15, 27]. The decoupling of capillary from myocyte precipitates an imbalance in oxygen and nutrients supply, resulting in microstructure derangements, fibrosis, and abnormal microvasculature. The hemodynamic factors include attenuation of vascular regulatory mechanisms, external compression of vessels, and retrograde coronary blood flow [9, 27, 28, 29]. The dynamic interplay between abnormal coronary blood flow and myocardial ischemia exacerbates myocardial perfusion abnormalities, which may further exacerbate fibrosis and microcirculation damage [10, 28]. Overall, the interrelationship between disarray, perfusion, and myocardial mechanics explains the complex link between microstructural alterations, hemodynamic changes and LV hypertrophy. Nonetheless, the underlying mechanisms remain intricate and are not yet fully understood.

Understanding of CMD in the context of HCM has been evolving for some time. Camici et al., initially described an inadequate increase in myocardial blood flow (MBF) following intravenous dipyridamole in the majority of HCM patients studied using PET, suggesting that microcirculatory impairment might not be an uncommon phenomenon in HCM patients [30]. Building on this, the team further established through PET that the severity of CMD is a robust predictor of exacerbation and mortality in HCM [13]. Importantly, this landmark research emphasized that significant CMD can be present in individuals with mild or no symptoms, potentially indicating the condition years before any clinical decline [13]. Petersen et al., using CMR to measure MBF in HCM patients at rest and during hyperemia, discovered a reduced vasodilatory response in these patients, especially in the endocardium, which was proportional to the severity of the hypertrophy [9]. Subsequently, various researchers improved CMR techniques, confirming not only the prevalence of CMD in HCM patients but also its association with myocardial fibrosis, highlighting its predictive value for adverse outcomes such as mortality and cardiac dysfunction and underscoring the essential role of microcirculatory assessment in HCM patient management [10, 11, 16, 31]. Consistent with aforementioned studies, the majority of patients included in our study had mild symptoms; however, nearly one‐third of patients had CMD in the LAD zone, which was associated with a worse long‐term prognosis.

Another notable observation is that, based on the criterion of angio‐IMR > 25 as the cutoff for CMD, CMD is detectable in all three coronary vessels. This observation aligns with previous research demonstrating that the coronary vasodilatory reserve impairment can occur in both hypertrophic and in nonhypertrophic myocardium regions [1, 6, 7]. One study suggested that CMD can develop early in the pathogenesis of HCM and is caused by abnormal formation of myocardial capillaries rather than regression [32], which likely explains the widespread microcirculatory damage observed in HCM patients [30]. However, in studies assessing CMD in relation to prognosis, whether through histological or imaging methods, researchers have mostly focused on areas of myocardial hypertrophy regions [9, 10, 11, 13, 14]. In agreement with these findings, we observed that CMD in LAD zone is associated with long‐term prognosis in HCM patients. A plausible mechanism is that, beyond microvascular structural abnormalities and functional impairments, increased oxygen demand from hypertrophic myocardium coupled with impaired oxygen delivery caused by CMD creates a severe oxygen supply–demand imbalance. This imbalance exacerbates ischemia in the hypertrophic myocardium regions, worsening clinical symptoms and precipitating adverse outcomes. Given that the interventricular septum, particularly its basal segment, represents the most common site of hypertrophy in HCM, CMD in the LAD territory may exert disproportionately greater effects. Future combined CMR and angio‐IMR analysis may further elucidate the differences prognostic implications of CMD across various coronary territories.

Coronary interventional physiological techniques, such as CFR and IMR, have emerged as indispensable tools for evaluating the coronary microvascular function, playing a pivotal role in the diagnosis and therapeutic decision‐making process for individuals with coronary artery disease [33, 34, 35, 36]. Several small cohort studies have also used these techniques to assess the microvascular function of HCM patients and verify the relationship between their microvascular function and prognosis [14, 37, 38]. Regrettably, although these technologies may offer valuable insights into the microvascular function of HCM patients and guide treatment, they have not been routinely applied in the assessment of HCM patients due to the complexity of the procedures, the use of vasodilator drugs, and the discomfort experienced during the tests. To overcome these issues, this study innovatively utilized the recently emerged angio‐IMR technology, developed through computational fluid dynamics, to conduct a comprehensive assessment of coronary microvascular function in all HCM patients undergoing coronary angiography, potentially contributing to precise management of HCM patients [19, 39, 40].

The present study has certain limitations. First, the retrospective design inherently poses risk of selection bias, and so on. To mitigate potential biases, we enrolled all consecutive patients fulfilling the predefined inclusion criteria and systematically monitored their postdischarge outcomes at regular intervals. Second, the patients included in this study had relatively mild symptoms, and the proportion of LVOT obstructive patients was lower than in other studies [41]. This could be due to the fact that some HCM patients with LVOT obstruction underwent SRT upon admission, and as a result, they were not candidates for comprehensive coronary angiography. Our subgroup analysis revealed that the LAD angio‐IMR holds prognostic value for HCM patients, regardless of whether LVOT obstruction is present or not. Third, our study focused on hard endpoints associated with HCM, but had a limited assessment of the relief of clinical symptoms such as angina in patients. This limitation somewhat restricts the generalizability of our findings, which is primarily due to insufficient consideration during the initial study design phase.

In conclusion, this study establishes that LAD angio‐IMR serves as an independent risk factor for the occurrence of MACE in HCM patients. Concurrently measurement the LAD angio‐IMR based on angiographic images during coronary angiography for HCM patients can aid in the prompt identification of those at higher risk, offering a novel approach to refine the management strategies for HCM patients.

## 4 Materials and Methods

### 4.1 Study Protocol

This large‐scale trial was conducted at two medical centers: The Second Affiliated Hospital, School of Medicine, Zhejiang University in Hangzhou, China and Ningbo Medical Center Lihuili Hospital, Ningbo University, in Ningbo, China (NCT06434415).

This study retrospectively enrolled patients aged 18 years and older, diagnosed with HCM at 2 medical centers between January 1, 2017, and March 31, 2023, who underwent coronary angiography evaluation due to symptomatic indications. Diagnosing HCM follows established protocols, relying predominantly on echocardiography or CMR to detect LV wall thickness of at least 15 mm, or over 13 mm accompanied by a positive genetic test or a familial predisposition, after excluding other possible cardiac or systemic conditions [1, 42]. Patients were excluded from this study if they: underwent concurrent PCI; had significant clinical comorbidities, including severe aortic stenosis or third‐degree atrioventricular block, malignant tumors, severe hepatic or renal dysfunction, or were status post‐transplant; had previously received SRT or ICD implantation; had ineligible coronary angiographic images.

### 4.2 Echocardiographic Studies, Angiographic Analysis, and Angio‐IMR Computation

Each patient received a baseline echocardiogram, followed by invasive coronary angiography, with both procedures adhering to standardized clinical standards and local best practices. Selection of ancillary medications such as calcium channel antagonists, beta‐blockers, and so forth, was determined by the attending physician's clinical judgment, guided by current evidence‐based recommendations. Calculations of angio‐IMR were performed with specialized software (AccuIMR, Version 1.0, ArteryFlow Technology, Hangzhou, China) as previously outlined, and the assessment process was blinded and managed by an independent core laboratory [19, 39, 40]. Angio‐IMR measurements were obtained for all three principal coronary vessels in each patient.

### 4.3 Endpoint and Follow‐Up

The primary endpoint was major advance cardiovascular event (MACE) throughout the follow‐up period, defined as a composite of cardiovascular death, heart failure readmission, life‐threatening ventricular arrhythmias requiring ICD implantation, SRT, or new‐onset stroke. Clinical events were adjudicated according to the criteria established by the Academic Research Consortium report [43]. All deaths were considered to be of cardiovascular death unless there was an established alternative cause. Heart failure readmission was defined by new or worsening signs and symptoms corroborated by imaging evidence or elevated BNP/NT‐proBNP levels. Event adjudication was conducted independently by two senior cardiologists, with discrepancies subjected to a consensus resolution protocol.

The follow‐up protocol encompassed clinic visits, medical record audits and structured telephone interviews, concluding with a final data cutoff on March 30, 2024.

### 4.4 Statistical Analysis

Continuous variables distribution characteristics were evaluated through the Kolmogorov–Smirnov test, along with graphical assessment using Q–Q plots and histograms. These variables were presented as mean ± SD or median with IQR, and comparisons were made using either the Student's *t*‐test or the Mann–Whitney *U*‐test, depending on the data's distribution. Categorical variables were reported as counts with percentages, with group differences examined via chi‐square test. The optimal angio‐IMR cutoff was identified through maximally selected log‐rank statistics [44]. The cumulative incidence of clinical events was estimated using the cumulative incidence function approach, and any differences in cumulative incidence were assessed by the Fine–Gray test [45]. Cox proportional hazard model was utilized to identify independent predictors of the primary endpoint. Proportional hazards assumption was examined using log‐negative log plots. Covariates with *p* < 0.1 in univariate analysis or clinically relevant were included in the multivariable Cox proportional hazard model. Subgroup analyses were performed with interaction testing to evaluate heterogeneity across patient subgroups. Propensity score matching (PSM) was implemented to adjust for baseline covariate imbalances. Statistical significance was established at a two‐tailed *p* value < 0.05. Analyses were executed utilizing SPSS (Version 26, IBM Corp., NY, USA) and R software (Version 4.1.2, R Foundation for Statistical Computing, Vienna, Austria).

## Author Contributions

Yuxuan Zhang, Rui Ji, and Shuxin Lei led the study's design, methodology, data work (acquisition/analysis), investigation, and initial drafting. Jingnan Pan handled data acquisition and interpretation. Zining Chen, Shitian Guo, Delong Chen, Abuduwufuer Yidilisi, Jiacheng Fang, Yiyue Zheng, and Xinyi Zhang participated in data collection and assisted in manuscript revision. Chi Liu and Jiniu Huang provided guidance on statistical analysis and methodological approaches. Yumeng Hu, and Jianping Xiang contributed to the technical support for the study. Xiaojie Xie, Jian'an Wang, and Jun Jiang were responsible for the overall supervision of the study, revised the manuscript, and ensured the integrity of the research. The final version of the manuscript has been reviewed and endorsed by all authors.

## Ethics Statement

This study obtained approval from the institutional review boards of both participating sites (approval number: 2022‐0940) and was conducted in full adherence to the Declaration of Helsinki and Good Clinical Practice guidelines. Given the retrospective design of the study, which utilized anonymized data, the committee exempted the requirement for individual participant consent. Furthermore, the study has been registered on ClinicalTrials.gov (NCT05884463).

## Conflicts of Interest

Dr. Jianping Xiang is principal scientist of ArteryFlow Technology. Mr. Yumeng Hu is the head of cardiac research of ArteryFlow Technology. All remaining authors declare no conflicts of interest.

## Supporting information



Supporting Information

## Data Availability

All data may be shared by the corresponding author after approval, provided that a valid reason is given for the request.
