# Supplementary material for: Prognostic Value of Coronary Angiography‐Derived Index of Microvascular Resistance in Patients With Hypertrophic Cardiomyopathy
Source: MedComm (2020). 2025 Jul 25;6(8):e70289. doi: 10.1002/mco2.70289 (PMC12290301; doi:10.1002/mco2.70289)
Supplement: Supplementary file 1 — Supporting Information [file MCO2-6-e70289-s001.docx]

**Prognostic Value of Coronary Angiography-derived Index of Microvascular Resistance in Patients with** **Hypertrophic Cardiomyopathy**

Yuxuan Zhang, MD^1,2,3,4#^; Rui Ji, MD^1,2,3,4#^; Shuxin Lei, MD^1,2,3,4#^; Jingnan Pan, MD^5^; Zining Chen, MD^1,2,3,4^; Shitian Guo, MD^1,2,3,4^; Delong Chen, MD^1,2,3,4^; Abuduwufuer Yidilisi, MD^1,2,3,4^; Jiacheng Fang, MD^1,2,3,4^; Yiyue Zheng, MD^1,2,3,4^; Xinyi Zhang, MD^1,2,3,4^;Chi Liu, PhD^1,2,3,4^; Jiniu Huang, PhD^1,2,3,4^;Yumeng Hu, MS^6^; Jianping Xiang, PhD^6^; Xiaojie Xie, MD, PhD^1,2,3,4*^; Jian’an Wang, MD, PhD^1,2,3,4*^; Jun Jiang, MD, PhD^1,2,3,4*^

#YZ, RJ and SL contributed equally as first authors.

Affiliations:

^1^Department of Cardiology of The Second Affiliated Hospital, School of Medicine, Zhejiang University, Hangzhou, China

^2^State Key Laboratory of Transvascular Implantation Devices, Hangzhou, China

^3^Cardiovascular Key Laboratory Zhejiang Province, Hangzhou, China

^4^Transvascular Implant Instrument Research Institute, The Second Affiliated Hospital Zhejiang University School of Medicine, Binjiang District, Hang Zhou 310053, China

^5^Department of Cardiology, Ningbo Medical Center Lihuili Hospital, Ningbo University, Ningbo, Zhejiang, China.

^6^ArteryFlow Technology Co., Ltd., Hangzhou, China

Address for Correspondence:

Xiaojie Xie ([xiexj@zju.edu.cn](mailto:xiexj@zju.edu.cn)); Jian'an Wang ([wangjianan111@zju.edu.cn](mailto:wangjianan111@zju.edu.cn)); Jun Jiang ([jiang-jun@zju.edu.cn](mailto:jiang-jun@zju.edu.cn))

Supplemental Appendix

|  | Page |
| --- | --- |
| Table S1: Univariable Cox Proportional Survival Analysis for MACE in HCM Patients | 3 |
| Table S2: Baseline Clinical Characteristics of Included Patients after Propensity Score Matching | 4 |
| Figure S1. The Propensity Score Matching Analysis. | 5 |
| Figure S2. Primary Clinical Outcomes in HCM Patients According to Different Conditions | 6 |

Table S1: Univariable Cox Proportional Survival Analysis for MACE in HCM Patients

| Covariates | HR (95% CI) | P value |
| --- | --- | --- |
| Demographics | | |
| Male sex | 0.759 (0.461-1.250) | 0.278 |
| BMI | 0.978 (0.907-1.056) | 0.573 |
| Smoking | 0.912 (0.537-1.551) | 0.735 |
| Alcohol intake | 0.785 (0.426-1.446) | 0.437 |
| Cardiovascular risk factors | | |
| Diabetes mellitus | 0.820 (0.391-1.723) | 0.601 |
| Hypertension | 0.977 (0.596-1.601) | 0.925 |
| Dyslipidemia | 1.224 (0.738-2.030) | 0.433 |
| CAD | 0.802 (0.427-1.504) | 0.491 |
| Family history | | |
| Hypertrophic cardiomyopathy | 0.493 (0.068- 3.558) | 0.483 |
| Sudden death from cardiac causes | ﹤0.001 (0.000-∞) | 0.996 |
| Echocardiographic indices | | |
| LVOT obstruction (≥30 mm Hg) | 1.215 (0.719-2.053) | 0.468 |
| AO-STJ | 1.503 (0.756-2.990) | 0.245 |
| LVIDs | 1.338 (0.806-2.220) | 0.260 |
| Angiography-derived indices | | |
| LAD angio-FFR | 0.125 (0.002-7.637) | 0.321 |
| LAD MB | 0.631 (0.321-1.242) | 0.183 |
| Medical treatment | | |
| CCB | 1.152 (0.693-1.916) | 0.585 |
| ACEI/ARB | 1.005 (0.609-1.657) | 0.985 |
| Amiodarone | 2.135 (0.855-5.330) | 0.104 |
| Statin | 0.942 (0.532-1.665) | 0.836 |

HR hazard ratio; CI confidence interval; other abbreviations as in Table 1.

Table S2: Baseline Clinical Characteristics of Included Patients after Propensity Score Matching

|  | High angio-IMR (n=85) | Low angio-IMR (n=85) | P value |
| --- | --- | --- | --- |
| Demographics | | | |
| Age, year | 59±12 | 59±11 | 0.828 |
| Male sex, n (%) | 55 (64.7) | 56 (65.9) | 0.872 |
| Alcohol intake, n (%) | 23 (27.1) | 24 (28.2) | 0.864 |
| NYHA functional class, no. (%) | | | 0.563 |
| Ⅰ | 27 (31.8) | 25 (29.4) |  |
| Ⅱ | 40 (47.1) | 48 (56.5) |  |
| Ⅲ | 14 (16.5) | 9 (10.6) |  |
| Ⅳ | 4 (4.7) | 3 (3.5) |  |
| Echocardiographic indices | | | |
| LV obstruction (≥30 mm Hg), n (%) | 25 (29.4) | 26 (30.6) | 0.867 |
| EF | 66±8 | 66±8 | 0.901 |
| LVIDd | 4.47±0.59 | 4.53±0.57 | 0.505 |
| LVIDs | 2.82±0.44 | 2.84±0.43 | 0.663 |
| LVM | 234.81±78.88 | 233.74±85.33 | 0.932 |
| Angiography-derived indices in LAD | | | |
| Angio-FFR | 0.94±0.03 | 0.94±0.02 | 0.570 |
| Medical treatment | | | |
| β-blocker | 70 (82.4) | 74 (87.1) | 0.394 |
| Antiplatelet | 53 (62.4) | 51 (60.0) | 0.753 |
| Anticoagulant | 8 (9.4) | 5 (5.9) | 0.387 |

Abbreviations as in Table 1.

Figure S1. The Propensity Score Matching Analysis.


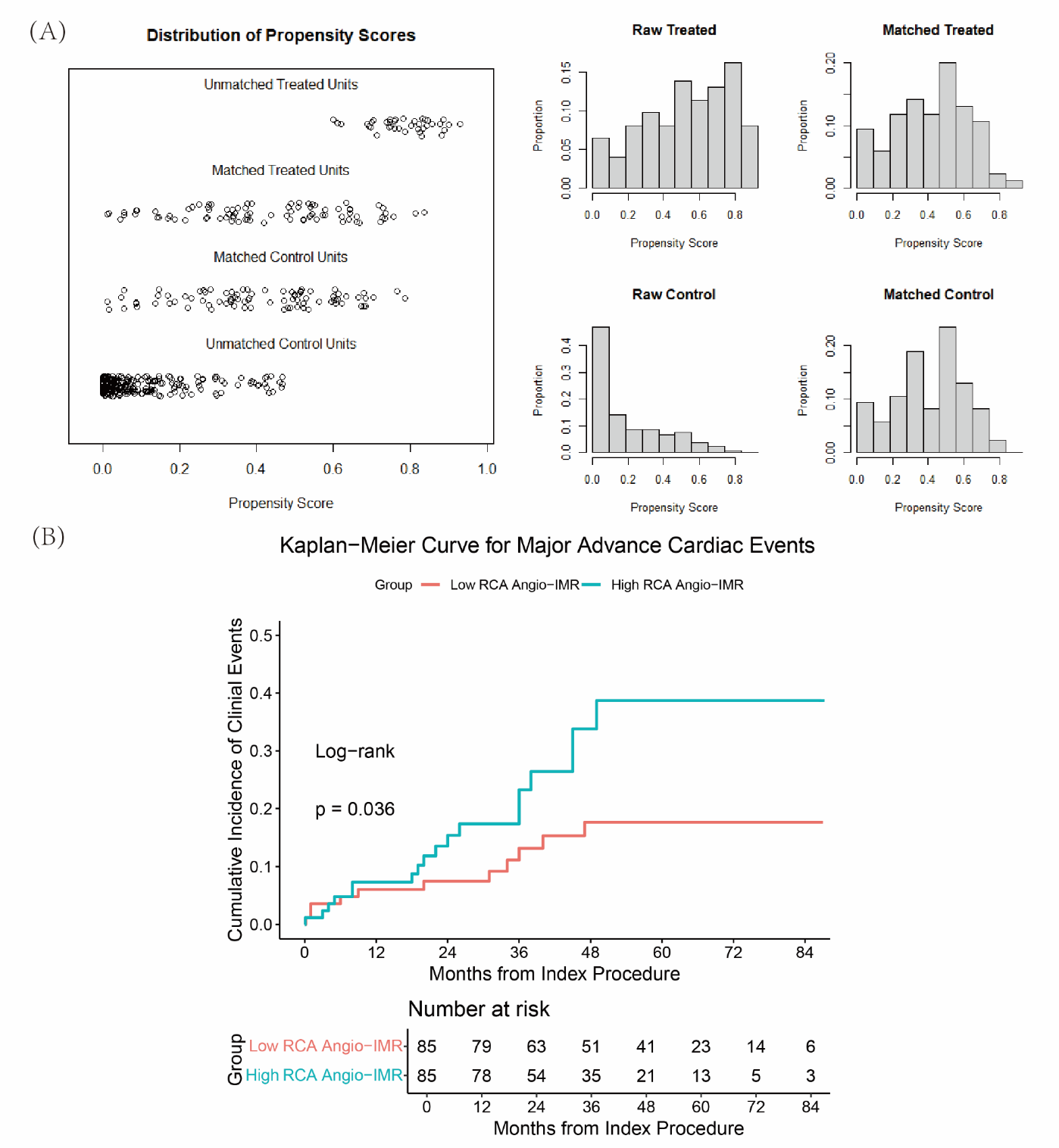


(A) Distribution after matching. The propensity score matching calculations included the following variables: age, sex, alcohol intake, NYHA functional class, LV obstruction, EF, LVIDd, LVIDs, LVM, angio-FFR in LAD and medical treatment including β-blocker, antiplatelet, anticoagulant. (B) Primary clinical outcomes in HCM patients according to LAD angio-IMR after propensity score matching. Abbreviations as in Table 1.

Figure S2. Primary Clinical Outcomes in HCM Patients According to Different Conditions


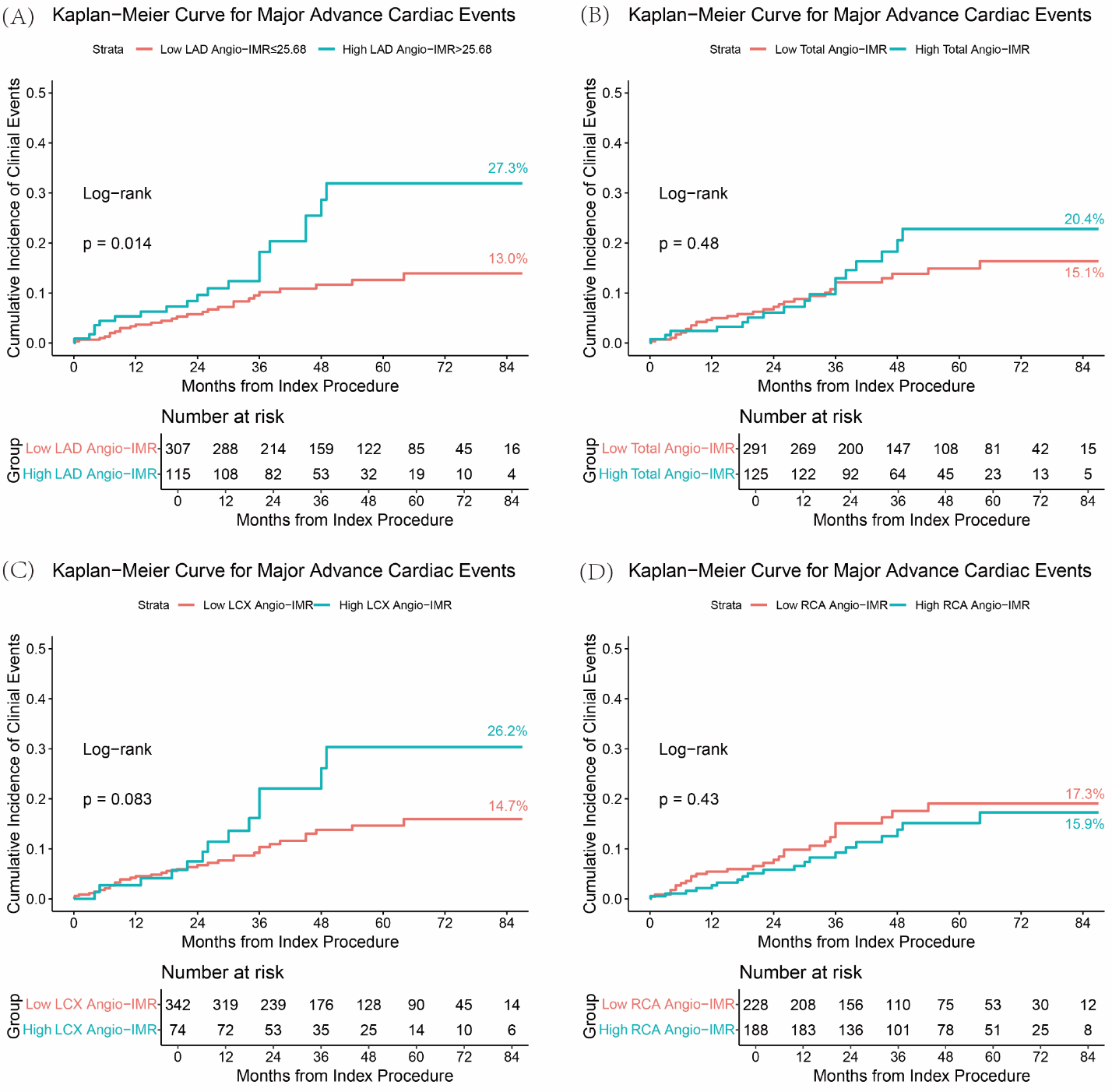


(A) LAD >25.68, (B) Total angio-IMR, (C) LCX angio-IMR and (D)RCA angio-IMR. LCX left circumflex artery; RCA right coronary artery; other abbreviations as in Figure 1.
